# Virtual and augmented reality in the vestibular rehabilitation of peripheral vestibular disorders: systematic review and meta-analysis

**DOI:** 10.1038/s41598-021-97370-9

**Published:** 2021-09-08

**Authors:** Austin Heffernan, Mohammed Abdelmalek, Desmond A. Nunez

**Affiliations:** 1 Division of Otolaryngology – Head and Neck Surgery, Department of Surgery, University of British Columbia, 2775 Laurel Street, 4th floor, Vancouver, BC V5Z 1M9 Canada; 2 Division of Otolaryngology – Head and Neck Surgery, Department of Surgery, Vancouver General Hospital, Vancouver, BC Canada

**Keywords:** Neurological disorders, Rehabilitation, Diseases, Medical research

## Abstract

Vestibular rehabilitation therapy is an established treatment for patients with vestibular dysfunction. Virtual reality (VR) and augmented reality (AR) can be utilised in vestibular rehabilitation. Evidence of the efficacy of VR and AR delivered rehabilitation in patients with peripheral vestibular disorders is reviewed. MEDLINE, EMBASE, CENTRAL, CINAHL, PsychInfo, PsychBITE, OTSeeker, Ei Compendex, IEE, Clinical trials.gov and WebofScience databases were searched. Reduction in vestibular dysfunction symptoms 0–3 months post-intervention was the primary outcome. Secondary outcomes included long-term symptom improvement and side effects. Risk of bias assessment and meta analyses were planned. Five studies meeting eligibility criteria were included. Dizziness Handicap Inventory (DHI) scores 0–3 months post-intervention were reported by four studies. Meta-analysis identified a 1.13 (95% CI, − 1.74, − 0.52) standardized mean difference reduction in DHI in VR and AR treated patients compared to controls. Side effects reported by two studies were reduced by week four of VR intervention. Bias assessment identified DHI scores and side effects to be at high risk or of some concern. Adjunct VR interventions reduced patient DHI significantly more than vestibular rehabilitation alone 0–3 months post-intervention in adult patients diagnosed with unilateral vestibular disease. High quality studies are needed.

## Introduction

Dizziness is a common complaint affecting up to 23% of the population at any time in a first world setting^1^. In publicly funded healthcare systems 0.8–1.7% of general practitioner attendances are for symptoms of dizziness or vertigo^2,3^, 9–13% of whom are referred to other specialists such as neurologists, cardiologists and otolaryngologists^3,4^. Disorders of the vestibular system are identified in 50–65% of patients seen in specialist dizziness clinics^5,6^. The vestibular system consists of sensory organs, cortical and subcortical structures that contribute to balance alongside proprioception and vision. The vestibular apparatus of the inner ear is the primary input for the vestibular system and relays information on head position and motion to the midbrain. This sensory information leads to adjustments in body movements and posture to maintain balance^7^. The vestibular system can be affected by a variety of peripheral vestibular disorders (PVD), including benign paroxysmal positional vertigo (BPV), Menière’s disease (MD), vestibular neuritis (VN) and post-traumatic vestibular dysfunction. Chronic dizziness symptoms can lead to symptoms of anxiety and depression^8,9^. Dizziness and its sequelae can create a dizziness handicap for symptomatic patients and carries a $64,929 lifetime burden for affected older adults^10^.

Vestibular rehabilitation is the main therapeutic option for many patients with dizziness. McDonnell and Hillier's^11^ Cochrane review concluded that there was moderate to strong evidence of its effectiveness in individuals with Unilateral Vestibular Hypofunction (UVH). Vestibular rehabilitation is an umbrella term that covers a range of exercise regimens from the generic such as Cooksey Cawthorne exercises to the customised^11^. The American Physical Therapy Association Clinical Practice Guidelines indicate that vestibular exercises are effective when compared to no or placebo exercises and that customized exercises are more effective than generic^12^. Vestibular rehabilitation can be done at home, in the clinic or in a combination of these settings. However, home exercises require the patient to be motivated to participate, which is an area where the playful activities utilized in virtual reality (VR) and augmented reality (AR) may add benefit. Seventy three percent of patients suffering from vestibular disease report more enjoyment and motivation with VR and AR interventions than vestibular rehabilitation^13^. Campbell et al.^14^ highlighted the importance of adherence to treatment in determining the benefit achieved through home based interventions aimed at preventing falls in the visually impaired. Interactive video gaming systems linked to an aerobic training regimen increase adherence and physical fitness attainment more than an aerobic training regimen alone^15^. Therefore, the use of VR/AR could improve vestibular rehabilitation compliance and overall outcomes^16,17^.

Virtual reality and AR are two ways of delivering potential reality type vestibular rehabilitative interventions. Virtual reality is defined as the immersion of the user in an interactive environment that mimics reality^18^. However, much of the current literature considers non-immersive commercially available video gaming systems (non-IGS) (ex. Wii Fit) to be VR. In an effort to focus on interventions that meet the true definition of virtual reality, non-IGS will be excluded from this systematic review. Immersive VR treats vestibular dysfunction by placing the subject in a simulated real world through two different strategies. One utilizes outpatient systems that use a head mounted display VR device and the second uses total body immersion inpatient systems such as the Immersive Rehabilitation Exercise System (Gesturetek Heath)^19^. In contrast to VR, AR augments the real-world environment instead of replacing it. Augmented reality adds to the subject’s real world sensory input through computer-generated sound, text and graphics that are projected onto the user’s natural visual and auditory fields^20^. Augmented reality platforms have been used to treat vestibular disorders using AR eyewear^21^.

Virtual reality’s utility in the treatment of vestibular dysfunction lies in the possibility of it achieving improved habituation, substitution, and adaptation through a more motivated vestibular rehabilitation^22,23^. Virtual reality has been studied as a rehabilitation intervention and been found to elicit improvements in a variety of PVDs^19,24^. VR delivered vestibular rehabilitation has to the best of our knowledge not been assessed in current guidelines on the management of patients with vestibular disorders possibly due to its relative novelty. Furthermore, the previously published systematic review on the efficacy of VR in vestibular rehabilitation was inconclusive^25^. We aim to synthesize the evidence to test the hypothesis that patients suffering from a PVD who have received VR or AR vestibular rehabilitation interventions experience the same improvement in dizziness as patients who received vestibular rehabilitation alone or a comparable control treatment.

The primary objective was to assess the benefits of VR and AR interventions for vestibular dysfunction symptoms (dizziness, vertigo, imbalance or other) in patients suffering from PVD within 3 months of treatment. Secondary objectives include the long term (> 3 months) benefits, post-intervention change in patient quality of life, determining the adverse events (harms) associated with use of VR or AR interventions and determining the number of patients who failed to complete treatment.

## Methods

This study is registered on the International prospective register of systematic reviews (PROSPERO) (CRD42020184674)^26^.

### Identification and selection of randomized controlled trials

**Figure 1:**
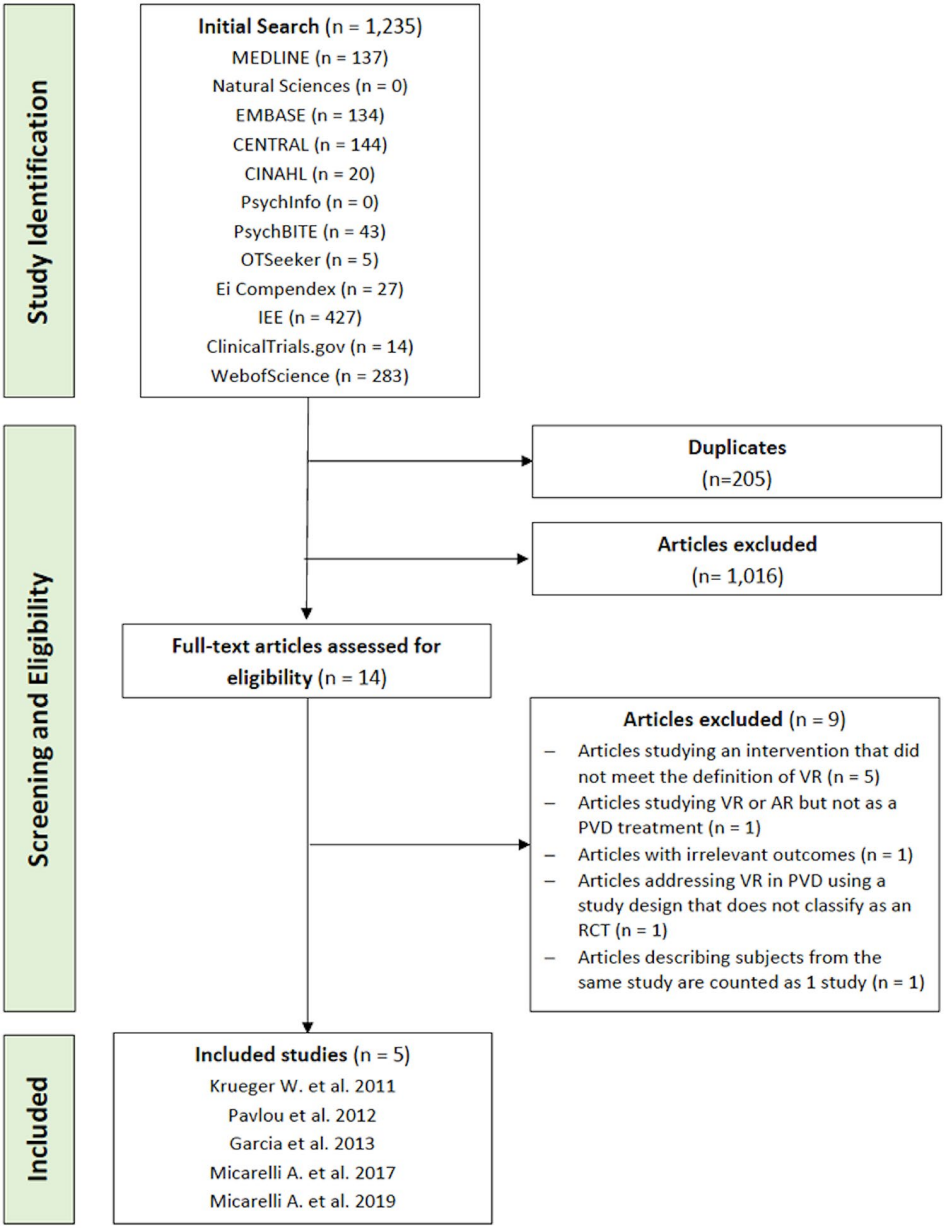
Flow of the article screening process for this systematic review. Flow chart was generated using Microsoft Word.

Randomized controlled trials (RCT) focusing on VR or AR vestibular rehabilitation interventions were gathered from MEDLINE, EMBASE, CENTRAL, CINAHL, PsychInfo, PsychBITE, OTSeeker, Ei Compendex, IEE, Clinical trials.gov and WebofScience (Fig. 1). The MEDLINE search strategy consisted of the following search terms and their synonyms combined by relevant Boolean operators: augmented reality, virtual reality, computer simulation, vestibular diseases, Meniere’s disease, motion sickness, vertigo, vestibular neuritis, mal de debarquement, postural balance, ototoxicity and semicircular canal dehiscence. This search strategy was peer reviewed and approved by two medical librarians. Subsequent database searches utilized this search strategy, but augmented search terms were used to fit database specifications. All databases were searched from their date of inception up to April 27th 2020 independently by two investigators.

Two investigators (AH and MA) screened papers from these selected databases based on their title, abstract, and content. Shortlisted papers were identified based on exclusion and inclusion criteria. Studies were excluded if they were animal studies, review articles, non-RCTs, quasi-controlled trials, case–control studies, cohort studies, cross-sectional studies, case reports, case series, letters, abstracts alone, or books. Patients needed to be diagnosed with a PVD. No restrictions were placed on study subjects age or sex; publication date or language except that the abstract and title must be in English. Only studies of VR or AR experimental interventions alone, or in combination with vestibular rehabilitation or diet were included. Control interventions included no treatment, medications and diet, diet alone, medications alone, placebo and medications, placebo alone, surgery alone, vestibular rehabilitation alone, or vestibular rehabilitation in combination with placebo, medications, diet, medications and diet, or surgery. Disagreements between AH and MA on study inclusion were arbitrated by the senior investigator (DN). If multiple reports on the same study were identified they were synthesized into one study report to reduce bias.

### Data extraction

AH and MA independently extracted data using the Cochrane Data Collection Form. Trial characteristics extracted included objective(s), design details, study funding sources and possible conflicts of interest. Participant data extracted included study sample demographics, setting, method of recruitment, number of subjects invited to participate, number of subjects randomized, number of missing and moved patients, and subgroups measured and reported. The extracted intervention data included description of interventions and control groups, resource requirements and compliance. Outcome data and analysis included measurement tools, imputation of missing data, power, intervention and control results, unit of analysis, statistical methods used and primary and secondary outcome measures. If multiple symptomatology scales were used, data from the most standardized scale was used. Discrepancies in data extraction were settled by consensus, or by the senior author if a consensus could not be reached.

### Assessment for bias

Following data extraction each included RCT was assessed for their risk of bias (RoB). The RoB was assessed using version 2 of the Cochrane-RoB tool for RCTs^27^. It includes bias from the randomization process, intervention deviations, missing outcome data, outcome measurement and selection of reported results. Each of the study outcomes of interest (i.e. Change in symptomatology at 0–3 months and > 3 months, and side effects) were independently RoB assessed according to Cochrane RoB2 tool recommendations. This was done independently by two investigators and disagreements were settled by the senior author. A study’s overall RoB was determined by the RoB in each domain. An overall low RoB requires that there is a low RoB in all domains. Some concerns of bias required there to not be a high RoB in any domain and for there to be at least one domain with some concerns of bias. Lastly, a study is judged to have an overall high RoB when the study is judged to have some concerns of bias in multiple domains or the study is judged to be at high RoB in at least one domain.

### Data analysis

Analysis of extracted data was completed using Cochrane RevMan 5.3^28^. Inter-trial statistical heterogeneity was assessed using the I^2^ test (low heterogeneity: < 25%, moderate heterogeneity: 25–75%, high heterogeneity: > 75%)^29^. When heterogeneity couldn’t be explained by characteristics of the RCTs, the data was incorporated into the meta-analysis using the random-effects model. When I^2^ < 50%, a fixed-effect model was used in the meta-analysis.

**Figure 2:**
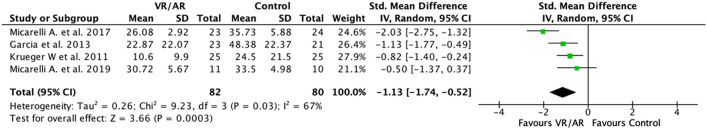
Post-intervention symptom index score (DHI) meta-analysis. SMD between VR/AR and control vestibular rehabilitation is listed. *DHI* dizziness handicap index, *SMD* standardized mean difference, *VR* virtual reality, *AR* augmented reality.

The summary statistics used in the meta-analysis were post-intervention mean values, and standard deviations. Standardized mean differences (SMD) were calculated for continuous data relevant to primary and secondary outcomes for change in Dizziness Handicap Index (DHI). The DHI score is a validated symptom index score created in 1990 that measures the functional, emotional and physical impacts of dizziness on the patient (Fig. 2)^30,31^. A summary intervention effect was then calculated as a weighted average with more weight given to values from studies with less variance and or more participants. Standard error of the summary intervention effect was used to derive a confidence interval (CI) and a *p* value. Significance cut off value (α) was set at a *p* value of 0.05. A random-effects model was planned and a forest plot display of results when studies did not use the same measure to estimate the same intervention effect.

### Sensitivity analysis

A sensitivity analysis was planned if a random effects model was adopted instead of a fixed effects model for the DHI-symptom index score–meta-analysis as recommendations on when to use each model is subject to much debate. The meta-analysis was undertaken twice, once using a fixed effects model and again using a random effects model to determine if and to what degree the result changed.

## Results

### Study selection

The search yielded 1235 articles from the twelve research databases reviewed. WHO ICTRP was not used due to restricted access. These 1235 articles were analysed by title and abstract which resulted in 1221 articles being excluded due to duplicates and the inclusion and exclusion criteria. Next, a full text screen was conducted where 9 articles were excluded due to the intervention not meeting the definition of VR (n = 5), the VR device not being used as a PVD treatment (n = 1), article reporting irrelevant outcomes (n = 1) and the study design not meeting RCT criteria (n = 1) (Fig. 1). Micarelli et al.^33^ and Viziano et al.^23^ reported initial and 12 month follow up reports on the same randomized controlled trial respectively. Their results were combined so they represented the same RCT, thus excluding the last of 9 articles. These two studies have been reported as Micerelli et al.^33^ in tables, figures and text. Therefore, a total of 5 RCT studies were eligible for this systematic review as listed in Fig. 1^23,32–36^.

### Description of studies

All five included studies were RCTs published from 2011 to 2019 that in total consisted of 107 control group participants and 97 intervention group participants (Table 1). Control and intervention groups were 53% female, had an average age ranging from 42 to 77 years of age and were suffering from a chronic PVD. The PVDs diagnosed in the studies were MD/endolymphatic hydrops (EH), neuritis, acoustic neuroma (AN), previous petrous surgery (PPS), previous cochlear surgery (PCS), Ramsay Hunt syndrome (RHS), labyrinth dysfunction (LD), benign positional vertigo (BPV), previous vestibular nerve section (PVNS) and motion intolerance (MI). Three studies focused on UVH^32,33,36^, one study focused on both UVH (n = 42) and bilateral vestibular hypofunction (BVH) (n = 1)^34^ and one study did not indicate PVD laterality^35^. Four of the five included RCTs compared the use of VR vestibular rehabilitation interventions to drugs, diet, at home exercises, standard vestibular rehabilitation (in clinic and at home) or static VR interventions (Table 1). Two of these four studies used VR interventions delivered with in-clinic devices while the remaining two studies used an at home head mounted display device. Only one of the five studies compared the use of AR during vestibular rehabilitation to vestibular rehabilitation without using AR (Table 1).

**Table 1:** Characteristics of the virtual or augmented reality protocol and study sample used in the included randomized controlled trials.

Author	Design	Study sample characteristics	Number of Volunteers		Characteristics of interventions	
			CG	IG	CG	IG
Garcia et al.^34^	RCT	Diagnosis: definite MD (UVH and BVH) DD > 6 months (%): 95.6% (CG), 90.5% (IG) Age: 47.90 (CG), 47.65 (IG) Sex: 60.9% F (CG), 66.7% F (IG)	23	21	Beta histidine (24 mg q12hr) Dietary recommendations: large breakfasts, light lunches, very light dinner, meals are less than 3 h apart, and patients refrained from refined sugar, coffee, alcohol and smoking	VR: BRU + VR goggles (in-clinic) Stimulus-enriched exercise on BRU performed in clinic for 45 min/session 2x/wk for 6 weeks Sessions = posturography, body balance rehabilitation and postural training games Co-intervention: beta histidine (24 mg q12hr) and dietary recommendations that mimic the control group
Krueger^35^	RCT	Diagnosis (% pop.): LD (48%), BPV (12%), LD + BPV(12%), MD/EH (12%) , PVNS (8%) or MI (4%) DD (mo) (mean ± SD): 29.2 ± 40.6 (CG), 28.6 ± 49.3 (IG) Age: 60.1 years (IG), 60.0 years (CG) Sex(%): 64% F (CG), 52% F (IG)	25	25	Vestibular rehabilitation in 30–60 min sessions without wearing the display device. Patients were informed to undergo as many sessions as needed to achieve improvements	AR: “User-worn see through display” (eyewear mounted visual display—displays artificial horizon) (in-clinic) Vestibular rehabilitation (30–60 min) while wearing display device
Micarelli et al.^33^	RCT	Diagnosis (% pop.): UVH [Neuritis (61%), AN (17%), PPV (9%), PCS (9%) and RHS (4%)] DD (mo) (mean ± SD): 9.37 ± 1.55 (CG), 9.91 ± 2.15 (IG) Age: 50.48 years (CG), 49.72 years (IG) Sex (F:M) = 46% F (CG), 39% F (IG)	24	23	Vestibular rehabilitation 30–45 min in clinic 2x/week for 4 weeks in combination with a 2x/day home exercise program for a total of 30–40 min/day	VR: Track Speed Racing 3D game run on 5.2’ display of a Phone (Lumia 930) accommodated into HMD ‘Revelation’ 3D VR Headset (Chinavasion) Vestibular rehabilitation in clinic 2x/wk for 4 weeks for 30–45 min in combination with 2x/day home exercise program for 30–40 min/day Performed game protocol with HMD device for 20 min/day at home
Micarelli et al.^32^ (older adults)	RCT	Diagnosis (% pop.): UVH [Neuritis (63%), AN (18%), PPS (9%), PCS (9%)] DD (mo) (mean ± SD): 198 ± 68.4 (CG), 206 ± 58.8 Age: 74.3 (CG), 76.9 (IG) Sex (F:M) = 50% F (CG), 55% F (IG)	24	23	4 weeks of vestibular rehabilitation and at home exercises	VR: Track Speed Racing 3D game run on 5.2’ display of a phone (Lumia 930) accommodated into HMD ‘Revelation’ 3D VR Headset (Chinavasion) 4 weeks of vestibular rehabilitation, home exercise and a track speed 3D racing game delivered through HMD based vestibular rehabilitation protocol 20 min/day at home
Pavlou et al.^36^	RCT	Diagnosis: Hx of acute onset vertigo with Hx indicative of VN DD (mo) (mean, range): 40.7 (6–88) (CG), 86 (50–156) (IG) Age: 42.1 (CG), 42 (IG) Sex: 36% F (CG), 60% F (IG)	11	5	Exposed to static 3D crowd in a street and asked to perform 9 exercises in sequence (repeated 2 × over 4 week period) Cawthorne-Cooksey exercises and general conditioning program at home simultaneously	VR device: ReaCTor Immersive Theatre (aka. CAVE)(in-clinic) Patients placed into a VR environment where computer generated humans walked at random rates per second and patients were instructed to perform nine exercises in sequence (45 min sessions, 2x/week for 4 weeks) Patients continued Cawthorne-Cooksey exercises and their general conditioning program at home

*AN* acoustic neuroma, *AR* augmented reality, *BPV* benign positional vertigo, *BRU* balance rehabilitation unit, *BVH* bilateral vestibular hypofunction, *CG* control group, *DD* disease duration, *EH* endolymphatic hydrops, *F* female, *HMD* head mounted display, *Hx* history, *IG* intervention group, *LD* labyrinth dysfunction, *M* male, *MD* Menieres disease, *MI* motion intolerance, *PCS* previous cochlear surgery, *PPS* previous petrous surgery, *PVNS* previous vestibular nerve section, *RCT* randomized controlled trial, *RHS*: Ramsay Hunt syndrome, *Sx* symptoms, *UVH* unilateral vestibular hypofunction, *VN* vestibular neuritis, *VR* virtual reality, *3D* three dimensions, *% pop* percentage of study sample.

### Risk of bias

Garcia et al.^34^ reported DHI scores that had a high RoB due to outcome measurement. Similarly, Krueger et al.’s^35^ study included DHI and motion sensitivity quotient (MSQ) scores which were both at a high RoB due to there being some concerns of bias in randomization, outcome measurement and reporting (Table 2).

**Table 2:** Risk of bias assessment results for all included randomized controlled trials.

RCT	Variable	RoB assessment domains					
		Randomization	Deviations from intervention	Missing outcome data	Outcome measurement	Reporting bias	Overall
Garcia et al.^34^	DHI	Low	Low	Low	High	Low	High
Krueger^35^	DHI	Some concerns	Low	Low	Some concerns	Some concerns	High
	MSQ	Some concerns	Low	Low	Some concerns	Some concerns	High
Micarelli et al.^33^	DHI	Low	Some concerns	High	Some concerns	Low	High
	ABC	Low	Some concerns	High	Some concerns	Low	High
	SSQ	Low	Some concerns	Low	High	Low	High
Micarelli et al.^32^	DHI	Low	Low	Low	Some concerns	Low	Some concerns
	ABC	Low	Low	Low	Some concerns	Low	Some concerns
	SSQ	Low	Low	Low	High	Low	High
Pavlou et al.^36^	SVQ	Some concerns	Low	Low	Some concerns	Low	High

*ABC* activities-specific balance confidence scale, *DHI* dizziness handicap index, *MSQ* motion sickness questionnaire, *RCT* randomized controlled trial, *RoB* risk of bias, *SSQ* simulator sickness questionnaire, *SVQ* situational vertigo questionnaire.

The RCT study reported by Micarelli et al.^33^ and Viziano et al.^23^ included DHI, Activities-specific Balance Confidence (ABC) scale and Simulator Sickness Questionnaire (SSQ) self-report measures. These DHI and ABC outcomes were both at high RoB due to some concerns of bias in measurement of the outcome and deviations from the intended interventions. The SSQ self-report data was also at a high RoB due to outcome measurement. The study published by Micarelli et al.^32^ also reported DHI, ABC and SSQ scores. The DHI and ABC outcomes had some concerns of bias due to outcome measurement, while SSQ was at a high RoB due to outcome measurement. Lastly, Pavlou et al.^36^ reported situational vertigo questionnaire (SVQ) scores, which had a high RoB due to some concerns of bias in outcome measurement and randomization (Table 2).

### Meta-analysis: effect of virtual and augmented reality on patient DHI 0–3 months post intervention

The DHI score was chosen as the main outcome measure for this meta-analysis as it was the most commonly reported patient symptomatology outcome among the included RCTs. Therefore, it meets the primary objective of a meta-analysis to amalgamate findings across several studies. Four studies reported DHI scores and these scores indicated that on average patients experienced moderate to severe functional impairment (DHI ≥ 31) prior to vestibular rehabilitation^37^. The I^2^ value and Chi^2^ *p* value were 67% and 0.03 respectively. The forest plot in Fig. 2 depicts Micarelli et al.^33^, Garcia et al.^34^ and Krueger et al.^35^ favoring VR and AR over controls for improving patient reported symptom control. Micarelli et al.^32^ also favored VR over control but this result was not significant. The combined SMD (−  1.13 [CI − 1.74, − 0.52]) significantly favors VR and AR over control interventions (Z = 3.66 [*p* = 0.0003]). Next, the causes of heterogeneity were investigated through subgroup analyses.

**Figure 3:**
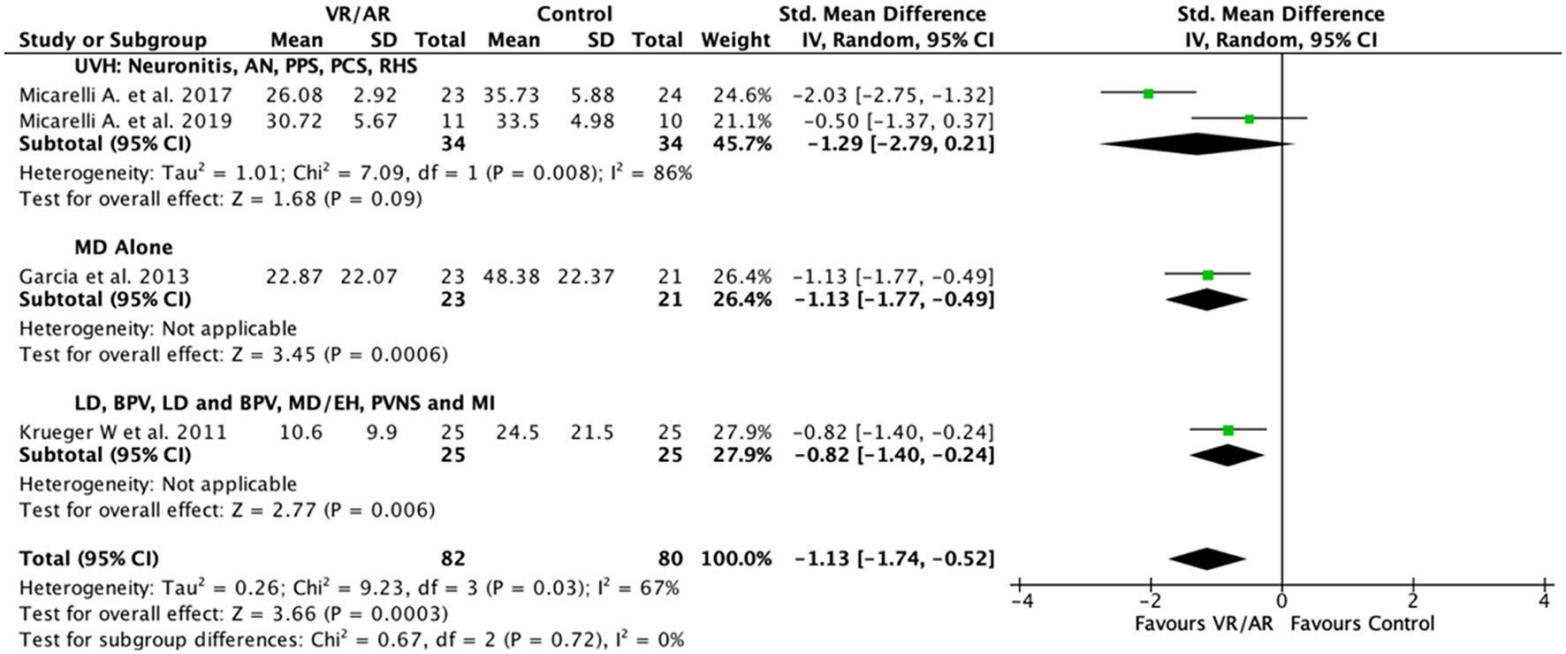
Post-intervention symptom index score (DHI) peripheral vestibular disorder subgroup analysis. SMDs between VR/AR and control vestibular rehabilitation for the group of UVH disorders studied by Micarelli et al.^33^ and Micarelli et al.^32^, MD alone and the vestibular disorders studied by Krueger^35^ are listed. *AN* acoustic neuroma, *AR* augmented reality, *BPV* benign positional vertigo, *DHI* dizziness handicap index, *EH* endolymphatic hydrops, *LD* labyrinth dysfunction, *MD* Meniere’s disease, *MI* motion intolerance, *PCS* previous cochlear surgery, *PPS* previous periosteal surgery, *PVD* peripheral vestibular disorder, *PVNS* previous vestibular nerve section, *RHS* Ramsay Hunt syndrome, *SMD* standardized mean differences, *UVH* unilateral vestibular hypofunction, *VR* virtual reality.

**Figure 4:**
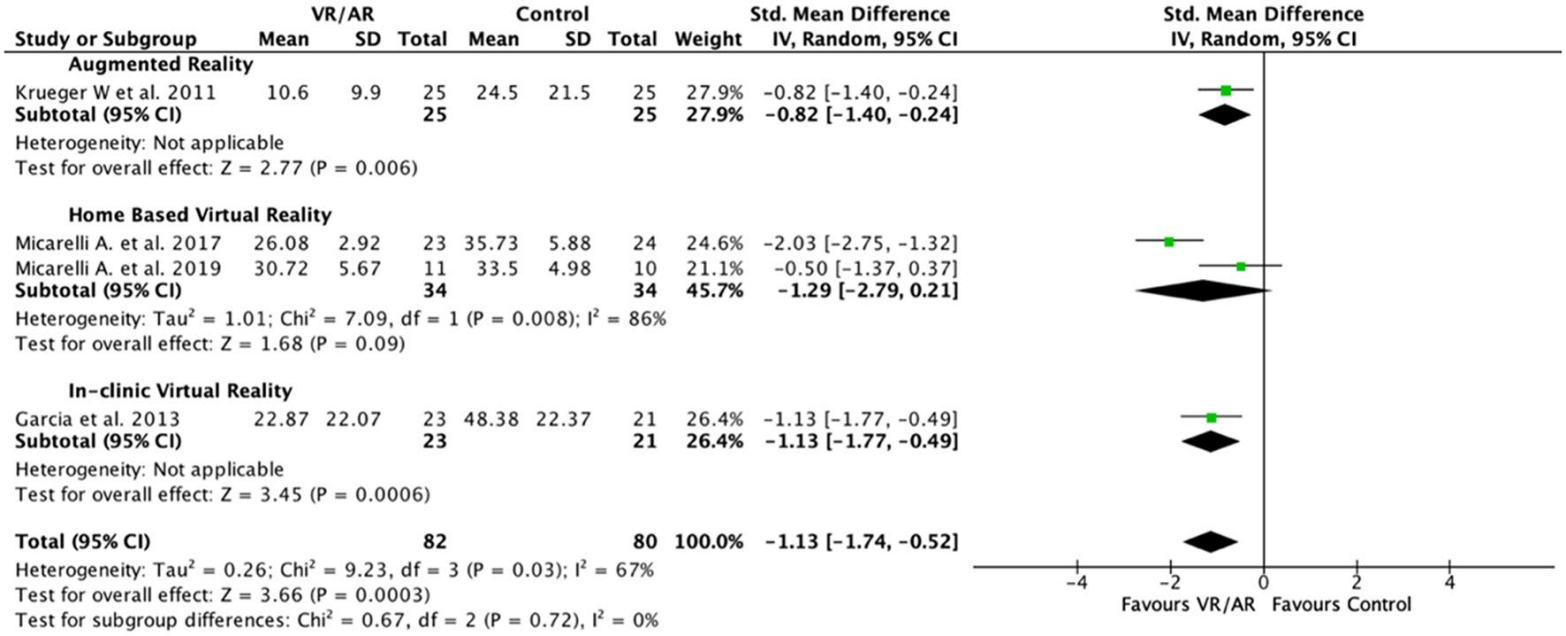
Post-intervention symptom index score (DHI) intervention type subgroup analysis. SMDs between VR/AR and control vestibular rehabilitation for AR, home based VR and in-clinic VR are listed. *DHI* dizziness handicap index, *SMD* standardized mean differences, *VR* virtual reality, *AR* augmented reality.

Two subgroup analyses were conducted, one based on PVD diagnosis and the second on intervention type. The direction of the effect of the VR interventions was the same across the PVD subgroups; UVH disorders (Neuronitis, AN, PPS, PCS and RHS) studied by Micarelli, unilateral and bilateral MD studied by Garcia and a group of PVDs of unstated laterality (LD, BPV, LD and BPV, MD/EH, PVNS and MI) studied by Krueger. Virtual reality was favored over controls for reducing dizziness handicap in patients diagnosed with MD (− 1.13 [− 1.77, − 0.49]) and UVH (− 1.29 [− 2.79, 0.211]) (Fig. 3). However, the latter did not reach significance due to Micarelli et al.^32^. Augmented reality was also significantly favored over controls for reducing dizziness handicap for patients diagnosed with various PVDs (− 0.82 [− 1.40, − 0.24]). Furthermore, the confidence interval estimates of the effect size for all VR and AR reality interventions overlapped in keeping with there being no significant heterogeneity due to sub-group diagnosis. The second subgroup analysis, type of intervention demonstrated no subgroup differences with identical direction of effect and similar overlap of effect size estimates in AR, and in clinic virtual reality (ICVR) both being favoured over control interventions (Fig. 4). Home based virtual reality (HBVR), however failed to demonstrate a significant advantage over control treatment (Fig. 4).

### Sensitivity analysis

It is recommended that a random-effects model be used when the same outcome is assessed using different measures across included studies or if heterogeneity in the data cannot be explained^38^. In this study all four analyzed studies measured self-perceived dizziness handicap using the same measure (DHI) and heterogeneity could not be explained by the type of PVD or intervention. Therefore, a sensitivity analysis was completed to determine if the decision to use a random effects model instead of a fixed effects model for the meta-analysis changed the findings. When using a fixed effects model for the DHI score meta-analysis the I^2^ and Chi^2^ *p* values changed from 67% and 0.03 to 79% and 0.002. Total SMD and 95% CI the values changed from − 1.13 [− 1.74, − 0.52] to − 8.74 [− 10.93, − 6.56], hence the direction of the effect favouring VR and AR interventions over control interventions remained congruent though the effect size was accentuated.

### Effect of virtual and augmented reality on balance confidence and dizziness 0–3 months post-intervention

Activities-specific balance confidence (ABC) scale is a validated measure of balance that was reported by two of the included RCTs^39,40^. One reported by Micarelli et al.^33^ and the second conducted by Marcarelli et al.^32^. Micarelli et al.^33^ indicated that 1 week post-intervention ABC scores for head mounted device (HMD) HBVR improved significantly (*p* = 0.0036) more than vestibular rehabilitation treatment. Micarelli et al.^32^ indicated that older adults completing HMD HBVR combined with vestibular rehabilitation had significant (*p* = 0.0082) improvements in ABC scores compared to vestibular rehabilitation alone 1 week after the intervention.

Dizziness was measured by Kreuger^35^ and Pavlou et al.^36^ using validated MSQ and SVQ scores respectively (Table 3)^41,42^. Kreuger^35^ detected a significant (*p* < 0.05) improvement from baseline for AR and control interventions, however there were no significant between group differences (Table 3). Despite this, the AR intervention reached these improvements after significantly (*p* ≤ 0.01) fewer rehabilitation sessions than vestibular rehabilitation alone. Pavlou et al.^36^ detected a significant (*p* = 0.001) between group difference in SVQ score change favouring dynamic (59.2% improvement) over static (1.6% improvement) VR interventions.

**Table 3:** Study results for quality of life, balance, dizziness, dynamic gait, vestibular ocular reflex gain and psychological outcome measures.

Author	Outcome	Instrument	Control		Experimental		Conclusions
			Pre (mean ± SD)	Post (mean ± SD)	Pre (mean ± SD)	Post (mean ± SD)	
Garcia et al.^34^	SIS	DHI	DHI: 52.67 ± 21.39	DHI: 48.38 ± 22.37	DHI: 57.57 ± 21.27	DHI: 22.87 ± 22.07	BRU with VR stimuli improved symptoms of dizziness and dizziness handicap in patients with Menieres disease
Krueger^35^	SIS Dizziness	DHI MSQ	DHI: 50.1 ± 31.2 MSQ: 34.9 ± 34.8	DHI: 24.5 ± 21.5 MSQ: 13.4 ± 18.8	DHI: 41.80 ± 18.5 MSQ: 26.5 ± 10.7	DHI: 10.6 ± 9.9 MSQ: 4.5 ± 6.3	AR group required less rehabilitation sessions to achieve the same outcomes as the control group
Micarelli et al.^33^	SIS Balance	DHI ABC	DHI: 55.91 ± 5.3 ABC: 65.08 ± 5.75	DHI: 35.73 ± 5.88 ABC: 73.21 ± 6.01	DHI: 56.6 ± 5.13 ABC: 64.78 ± 5.44	DHI: 26.08 ± 2.92 ABC: 78.56 ± 4.61	VR mixed with vestibular rehabilitation yielded better otoneurologic scores than vestibular rehabilitation alone
Micarelli et al.^32^ (older adults)	SIS Balance	DHI ABC	DHI: 61.16 ± 7.25 ABC: 64.91 ± 5.94	DHI: 33.5 ± 4.98 ABC: 72.41 ± 6.15	DHI: 64.00 ± 5.05 ABC: 62.54 ± 4.8	DHI: 30.72 ± 5.67 ABC: 71.36 ± 4.24	Home based HMD vestibular rehabilitation significantly improves symptoms of dizziness and balance confidence in older adults
Pavlou et al.^36^	Dizziness	SVQ	SVQ: 1.28 ± 0.75	SVQ: 1.26 ± 0.90	SVQ: 1.54 ± 0.50	SVQ: 0.63 ± 0.25	Vertigo symptoms improve when rehabilitation combines vestibular exercises with a dynamic VR environment

*ABC* activities specific balance confidence scale, *AR* augmented reality, *BRU* balance rehabilitation unit, *DHI* dizziness handicap score, *HMD* head mounted display, *MSQ* motion sensitivity quotient, *SVQ* situational vertigo questionnaire, *SD* standard deviation, *SIS* symptom index score, *VR* virtual reality.

### Effect of virtual and augmented reality on balance confidence and dizziness 3 or more months post-treatment

Viziano et al.^23^ reported a 12 month post-intervention ABC score for the HMD VR intervention that was significantly (*p* = 0.0022) more improved than with vestibular rehabilitation treatment alone.

### Side effects

Side effects of VR interventions were reported by Micarelli et al.^33^ and Micarelli et al.^32^ using the validated simulator sickness questionnaire (Table 4)^43,44^. Side effects were measured using the SSQ. In the HMD plus VR groups the nausea, oculomotor and disorientation symptoms associated with the VR intervention decreased over time. There was a significant reduction in nausea, oculomotor stress and disorientation scores from the first to fourth week of the VR rehabilitation program in both Micarelli et al.^33^ (*p* < 0.001) and Micarelli et al.^32^ (*p* < 0.05) studies (Table 4).

**Table 4:** Study results for virtual reality side effects.

Author	Outcome	Instrument		Control		Experiment		Significance
				Pre (mean ± SD)	Post (mean ± SD)	Pre (mean ± SD)	Post (mean ± SD)	
Micarelli et al.^33^	Side effects	SSQ	N	N/A	N/A	2.91 ± 0.73	1.52 ± 0.51	*p* < 0.001
			O			4.04 ± 0.70	2. 17 ± 0.51	*p* < 0.001
			D			3.78 ± 0.59	2.21 ± 0.51	*p* < 0.001
Micarelli et al.^32^	Side effects	SSQ	N	N/A	N/A	2.90 ± 0.70	1.36 ± 0.50	*p* < 0.05
			O			4.00 ± 0.63	2.09 ± 0.53	*p* < 0.05
			D			4.00 ± 0.77	1.90 ± 0.70	*p* < 0.05

*D* disorientation, *N* nausea, *O* oculomotor stress, *SD* standard deviation, *SSQ* simulator sickness questionnaire.

### Treatment adherence

Three of the five randomized controlled trials indicated a 100% adherence in both control and experimental intervention groups. However, one patient from the Garcia et al.^34^ study did not complete tests due to intense neurovegetative symptoms (97.7% adherence) and one patient from the Pavlou et al.^36^ did not complete the trial due to non-compliance (94.1% adherence). Assuming each study is weighted equally, the VR and AR vestibular rehabilitation interventions had an overall adherence of 98.4% over a 4–6 week study period. Unfortunately the exact duration of the Krueger^35^ RCT was not specified, however the number of visits required to achieve improvement was 4 visits with AR and 6 visits without AR.

## Discussion

In this systematic review, the effectiveness of VR and AR as vestibular rehabilitation interventions were assessed through their impact on patient symptomatology. The level of dizziness handicap experienced by study patients was assessed using the DHI. A meta-analysis was performed on DHI data that was reported by four of the five studies in this review. This meta-analysis was performed due to the consistency of the direction of the intervention effect in the individual studies and the moderate I^2^ assessed heterogeneity of 67% (Fig. 2). Moreover, it has been argued that heterogeneity in data is inevitable due to clinical and methodological differences consistently being present in meta analyses^45^.

Our meta-analysis supports adjunct VR as being superior to beta histidine, dietary recommendations, and vestibular rehabilitation alone including Cawthorne Cooksey home exercises for reducing patient DHI. This is in agreement with the only other systematic review on VR in vestibular rehabilitation^25^. However, the current systematic review provides the first meta-analysis of patient reported data derived exclusively from RCTs. This meta-analysis is of benefit in determining the effect size of adjunct VR and AR on vestibular symptoms, the predominant presenting feature of patients, compared to vestibular rehabilitation alone. Pre-registration of the current review’s protocol mitigates the risk of publication bias ^26^ and improves the robustness of the effect size findings.

The favourability of VR and AR interventions was not shown on sub-group analysis for HMD HBVR. This is likely due to the small sample size of the Marcarelli et al. (2019) RCT, and the age seniority (avg age: 75.6 years) of the participants studied. The latter is supported by Yan et al.^46^ who demonstrated that elderly patients (65–76 years) with vestibular neuritis, who constituted up to 63% of Micarelli’s study’s participants, respond less to vestibular rehabilitation than middle aged patients^46^. Previous literature supports an improvement in dizziness handicap by HMD VR devices that lasted for 1 week before relapsing^47^. Viziano et al.^23^ refutes early relapse as they demonstrated that 12 months after 4 weeks of vestibular rehabilitation plus HMD HBVR intervention the DHI scores remained significantly (*p* < 0.05) lower than pre intervention scores^23^. Based on the results of our subgroup analyses it can also be hypothesized that immersive ICVR induces the most substantial reduction in the dizziness handicap experienced by patients diagnosed with PVD. This is supported by Whitney et al.^37^, who demonstrated a reduction in DHI after 6 vestibular rehabilitation sessions (6 weeks) using an immersive ICVR that was similar to the system used by Garcia et al.^34,48^.

The 1.13 standardized mean difference in DHI score in favour of VR and AR interventions indicates a large intervention effect based on the rule of thumb that a 0.8 or greater standard deviation difference is consistent with a large treatment effect^49^. A second way of interpreting the VR and AR intervention effect size is to consider the minimally important difference (MID) in vestibular symptoms. The developers of the DHI scale determined that an 18-point score change represented a clinically important difference in vestibular symptoms^31^, hence this difference was adopted as the MID. The 1.13 SMD in DHI when converted into a DHI score using an estimate of the standard deviations of DHI scores across intervention and control groups in the studies in our meta-analysis as previously described^49^ yields a mean DHI difference of 14.07 (6.48, 21.67). This suggests that the VR and AR additional intervention effect is clinically unimportant. The two effect size estimates described result in diametrically opposed conclusions. In our opinion the VR/AR effect size is more likely to lie between these two extremes of a very large and no discernable effect at all. More high quality RCTs are required to increase the number of patients studied because of the inherently large heterogeneity in aetiology, symptom severity, patient ages and comorbidity relevant to vestibular rehabilitation to better refine determination of the effect size of VR and AR.

The ABC scale shares some similarities to the DHI as it evaluates patient restrictions by measuring the subjective level of balance confidence experienced in 16 daily activities. The use of HMD HBVR was shown to improve balance confidence in elderly and middle aged patients diagnosed with neuritis, AN, Ramsay–Hunt syndrome or a history of petrous or cochlear surgery^23,32,33^. This improvement was significantly (*p* < 0.05) greater than vestibular rehabilitation alone at 1 week and 12 months post-intervention^23,32,33^. However, results should be interpreted with caution because their respective studies did not control for patient physical activity, which has been shown to promote central vestibular compensation and improve vestibular rehabilitation outcomes in patients diagnosed with VN^50,51^. This is relevant because VN makes up the largest fraction of patients participating in the included RCTs that reported ABC data. Therefore, physical activity differences between VR and control groups could have impacted study results and should be controlled for in future studies.

The use of VR or AR also improves vertigo symptoms experienced by patients suffering from PVD. However not all VR elicits the same response. Pavlou et al.^36^ demonstrated that VR with a dynamic environment reduced vertigo symptoms (dizziness, giddiness, light-headedness or unsteadiness) approximately 50 fold more than static VR. Similarly, AR has shown utility in reducing dizziness symptoms. Krueger^35^ determined that using AR during vestibular rehabilitation allowed patients with moderate to severe functional impairment to reach the same improvement level in motion provoked dizziness as vestibular rehabilitation alone. However, AR reached this level in four visits instead of six, which is one visit less than the optimal number of treatment sessions needed to treat chronic UVH^52^. This is clinically relevant as it could reduce financial and social barriers (e.g. time commitment) to rehabilitation adherence, thereby improving rehabilitation compliance and patient outcomes^53^.

Side effects associated with VR interventions could be a barrier to the adoption of this technology. However, the two studies that reported VR side effects in this review noted that they occurred acutely but gradually reduced over time as patients habituate to the VR intervention^32,33,36,54^. These acute side effects are an intended part of vestibular rehabilitation interventions that aim to achieve habituation^55^. It is essential that patients are informed of these side effects and their purpose prior to implementing a VR vestibular rehabilitation intervention. Despite these side effects, 100% and 98.4% of participants were adherent to AR and VR interventions of 4–6 weeks duration. This could reflect the highly motivating nature of VR and AR interventions^13,56–58^.

Despite these positive results, this systematic review does have limitations. First, studies were also to be extracted from the WHO ICTRP database, but due to high COVID-19 search traffic the research team could not access the database. Moreover, the results of this systematic review and meta-analysis are more applicable to chronic unilateral vestibular disease rather than all PVD sufferers. This is because three of the five RCTs exclusively studied patients with UVH, and 98% of the patients in the fourth RCT studied were diagnosed with unilateral MD. Additionally, it is unsafe to extrapolate the review findings to patients with mild functional impairment secondary to UVH since the majority of patients in the included studies had moderate to severe vestibular functional impairment based on DHI. Similarly, only one AR RCT was included in this study, which means that there is lack of replicated RCT evidence to support benefit from AR in this patient group in the literature thus far. Lastly, 80% of the DHI, ABC, MSQ, SVQ and SSQ outcome measures were at a high RoB which weakens the review’s overall conclusions.

In conclusion, this meta-analysis indicates that VR vestibular rehabilitation improves DHI scores significantly more than vestibular rehabilitation alone 0–3 months post-intervention in patients diagnosed with moderate (DHI 31–60) to severe (DHI ≥ 61) vestibular impairment secondary to unilateral vestibular disease. To our knowledge this is the first level 1a evidenced^59^ meta-analysis supporting the use of adjunct VR in the vestibular rehabilitation of adult patients with unilateral vestibular disease. VR may also improve DHI and balance confidence (ABC) scores more than vestibular rehabilitation therapy at greater than 3 months post-intervention, however additional evidence is needed to support this statement. Side effects are also associated with VR sessions but these decreased significantly by the 4th week post-intervention. The validity of these conclusions are compromised by the high RoB in the data studied. Hence, while VR maybe a potential adjuvant to vestibular rehabilitation therapy alone for patients with unilateral vestibular disease additional high quality research is needed to support this assertion.

## Data Availability

The datasets used and analyzed during this study are available from the corresponding author upon request.
